# Detection of Antimicrobial Proteins/Peptides and Bacterial Proteins Involved in Antimicrobial Resistance in Raw Cow’s Milk from Different Breeds

**DOI:** 10.3390/antibiotics13090838

**Published:** 2024-09-03

**Authors:** Cristian Piras, Rosario De Fazio, Antonella Di Francesco, Francesca Oppedisano, Anna Antonella Spina, Vincenzo Cunsolo, Paola Roncada, Rainer Cramer, Domenico Britti

**Affiliations:** 1 Department of Health Sciences, Magna Græcia University of Catanzaro, 88100 Catanzaro, Italy; rosario.defazio@studenti.unicz.it (R.D.F.); foppedisano@unicz.it (F.O.); aa.spina@unicz.it (A.A.S.); roncada@unicz.it (P.R.); britti@unicz.it (D.B.); 2 Interdepartmental Center Veterinary Service for Human and Animal Health, University “Magna Graecia” of Catanzaro, CISVetSUA, 88100 Catanzaro, Italy; 3 Laboratory of Organic Mass Spectrometry, Department of Chemical Sciences, University of Catania, 95125 Catania, Italy; antonella.difrancesco@unict.it (A.D.F.); vcunsolo@unict.it (V.C.); 4 Institute of Research for Food Safety & Health (IRC-FSH), Department of Health Sciences, University of Catanzaro Magna Græcia, 88100 Catanzaro, Italy; 5 Department of Chemistry, University of Reading, Whiteknights, Reading RG6 6DX, UK; r.k.cramer@reading.ac.uk

**Keywords:** antimicrobial proteins, resistome proteins, bacterial consortia, bovine milk

## Abstract

Proteins involved in antibiotic resistance (resistome) and with antimicrobial activity are present in biological specimens. This study aims to explore the presence and abundance of antimicrobial peptides (AMPs) and resistome proteins in bovine milk from diverse breeds and from intensive (Pezzata rossa, Bruna alpina, and Frisona) and non-intensive farming (Podolica breeds). Liquid atmospheric pressure matrix-assisted laser desorption/ionization (LAP-MALDI) mass spectrometry (MS) profiling, bottom-up proteomics, and metaproteomics were used to comprehensively analyze milk samples from various bovine breeds in order to identify and characterize AMPs and to investigate resistome proteins. LAP-MALDI MS coupled with linear discriminant analysis (LDA) machine learning was employed as a rapid classification method for Podolica milk recognition against the milk of other bovine species. The results of the LAP-MALDI MS analysis of milk coupled with the linear discriminant analysis (LDA) demonstrate the potential of distinguishing between Podolica and control milk samples based on MS profiles. The classification accuracy achieved in the training set is 86% while it reaches 98.4% in the test set. Bottom-up proteomics revealed approximately 220 quantified bovine proteins (identified using the *Bos taurus* database), with cathelicidins and annexins exhibiting higher abundance levels in control cows (intensive farming breeds). On the other hand, the metaproteomics analysis highlighted the diversity within the milk’s microbial ecosystem with interesting results that may reflect the diverse environmental variables. The bottom-up proteomics data analysis using the Comprehensive Antibiotic Resistance Database (CARD) revealed beta-lactamases and tetracycline resistance proteins in both control and Podolica milk samples, with no relevant breed-specific differences observed.

## 1. Introduction

Milk is a complex and dynamic biological fluid which is important not only for its nutritional value but also for its role as a source of bioactive compounds with diverse functional properties. Among these compounds are antimicrobial peptides (AMPs) and resistome proteins, both of which are gaining increasing attention due to their potential significance in affecting human health and food safety [1]. AMPs play a crucial role in innate immunity by protecting the mammary gland and the infant gut against pathogenic microorganisms. Resistome genes code for a range of proteins involved in antimicrobial resistance (AMR) [2]. Their expression is not necessarily linked with anthropic antibiotic treatments, but might simply be generated by the competition between microorganisms for the same ecological niche [3,4]. However, the spread of AMR genes might be induced by pharmacological pressure in animal production [5,6]. Thus, monitoring the presence and abundance of resistome proteins in milk from different bovine breeds is of utmost importance, as it can provide insights into the potential risks associated with AMR genes or gene products in the environment.

AMPs, also known as host defense peptides, are endogenous molecules produced by the host with antimicrobial properties. They are synthesized by various cell types in the mammary gland, including epithelial cells and immune cells, or produced by the degradation of common milk proteins. The diverse array of AMPs found in milk helps to protect against infections and supports the development of a healthy immune system in neonates. Some well-known examples of milk-derived AMPs include lactoferrin, lysozyme, and casein-derived peptides [7]. Those types of defense mechanisms are part of the non-specific defense mechanisms and do not target species-specific bacterial structures as in the case of antibodies [8].

Bacterial resistome genes, on the other hand, code for all those proteins that are associated with antibacterial drug resistance. Beta-lactamases, for instance, are enzymes that catalyze the breakdown of beta-lactam antibiotics, a group that includes penicillins and cephalosporins. These enzymes effectively disarm these drugs by cleaving the crucial beta-lactam ring, rendering them inactive [9]. Aminoglycoside acetyltransferases, on the other hand, modify aminoglycoside antibiotics like gentamicin and kanamycin, rendering them ineffective by adding acetyl groups [10]. Similarly, tetracycline resistance proteins confer resistance to tetracycline antibiotics, which are widely used in treating various infections. These proteins work by actively pumping tetracyclines out of bacterial cells or by modifying the antibiotics themselves, diminishing their therapeutic potential [11].

One of the prominent bovine breeds recognized for its unique milk composition is the Podolica breed, which is widely spread in southern Italy. Podolica milk is known for its lower milk yields compared to other breeds and the slightly different composition of fatty acids that influence the overall nutritional value [12,13]. The Podolica cattle breed is commonly found in southern Italy, where it is typically raised in wild or semi-wild conditions in mountainous regions, with a lifespan of about 15 years [14]. These cattle have ancient European roots, descending from *Bos primigenius*, and are distinguished by their grey coats and long horns [15,16]. While Podolica cows are known for their low milk yields, the milk they produce is of exceptional quality, contributing to the production of highly valued and sought-after meat. A significant portion of this milk is used to make “Caciocavallo Podolico”, a cheese made exclusively from Podolica milk [16]. This milk boasts a high protein content (4.06%) and fat level (4.87%), and it is packed with various bioactive compounds, including peptides, vitamins C and E, carotenoids, and flavonoids, all known for their antioxidant properties. Additionally, it is abundant in unsaturated fatty acids (30%), particularly Omega 3 and Omega 6, which play a crucial role in maintaining skin hydration [17].

This study aims to provide a comprehensive examination of AMPs and resistome proteins in the milk of various bovine breeds. Mass spectral profiling using LAP-MALDI MS [18,19], proteomics, and metaproteomics were applied to (i) the classification of Podolica milk versus milk of other bovine species, (ii) the identification and characterization of the AMPs present in the milk of different bovine breeds with a focus on their diversity and abundance, and (iii) the investigation of resistome proteins.

## 2. Results

**Figure 1:**
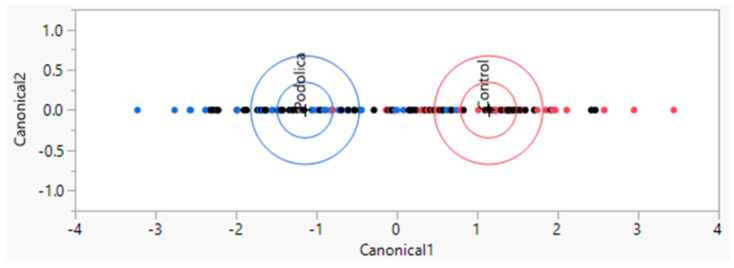
Linear discriminant analysis plot representing the LAP-MALDI MS classification of the Podolica (blue) versus control (red) milk samples. The control milk samples were obtained from three different breeds (Pezzata rossa, Bruna alpina, and Frisona).

LAP-MALDI MS profiling coupled with LDA on the training set allowed the creation of an LDA model (64 data points: 8 MALDI sample spot replicates for each biological replicate with 4 control and 4 Podolica biological replicates), which yielded the classification shown in Figure 1 with an accuracy of around 86%. Among the 32 control samples, 4 were classified as Podolica samples, and among the 32 Podolica samples, 5 were classified as control samples. The analysis of the test set (64 data points with the same distribution of technical and biological replicates as in the training set) yielded a correct classification percentage of 98.4%, classifying just 1 Podolica sample (out of 32) incorrectly as a control sample (Table 1).

**Figure 2:**
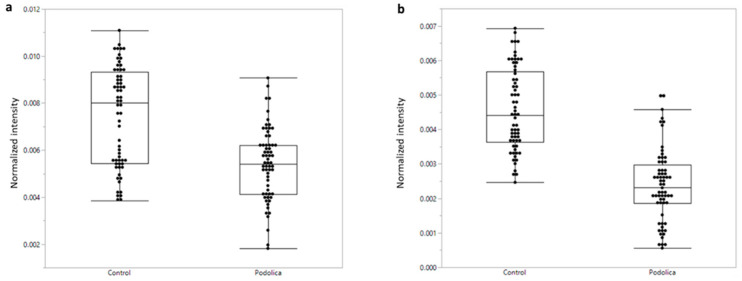
Normalized LAP-MALDI MS bin intensities (as fraction of the summed intensities of all bins) for the 1418–1419 (**a**) and 1576–1577 (**b**) mass bin. The ions of these mass bins are relevant for the breed classification, and the difference in their intensity between control and Podolica milk samples is statistically significant (*p* < 0.0001, Wilcoxon test) for both mass bins.

Two of the most influential ion species for the above training set classification are shown in Figure 2. Both ion species are from the same analyte charge distribution with 10 charges (Figure 2a) and 9 charges (Figure 2b), respectively. Multiplying the m/z bin values of the above ion species by their charge numbers and subtracting the number of applicable protons it is estimated that the average molecular weight of the neutral analyte molecule is between 14175 and 14180 Da.

For this bin data analysis, the samples of the training set were used, which led to a significant abundance difference between the Podolica samples and the samples of the other breeds.

**Figure 3:**
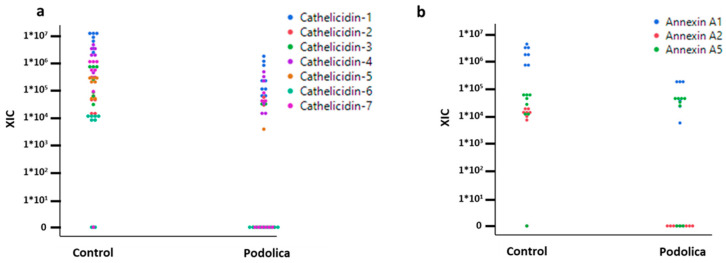
Differential representation of the summed eXtracted Ion Current (XIC) of all isotopic clusters associated with the identified amino acid sequence obtained through LFQ bottom-up proteomic analysis of cathelicidins (**a**) and annexins (**b**).

After the bacterial cell and somatic cell enrichment, the obtained samples were analyzed via bottom-up proteomics. The LC-MS/MS raw files were searched in *Bost taurus*, *bacteria*, and CARDs in separate searches (see the Methods Section). The LFQ analysis using MaxQuant and the *Bos taurus* database allowed the identification of around 220 proteins. Among those, as shown in Figure 3a,b, cathelicidins and annexins were differentially represented, showing a higher level of abundance in the milk of the control cows.

**Figure 4:**
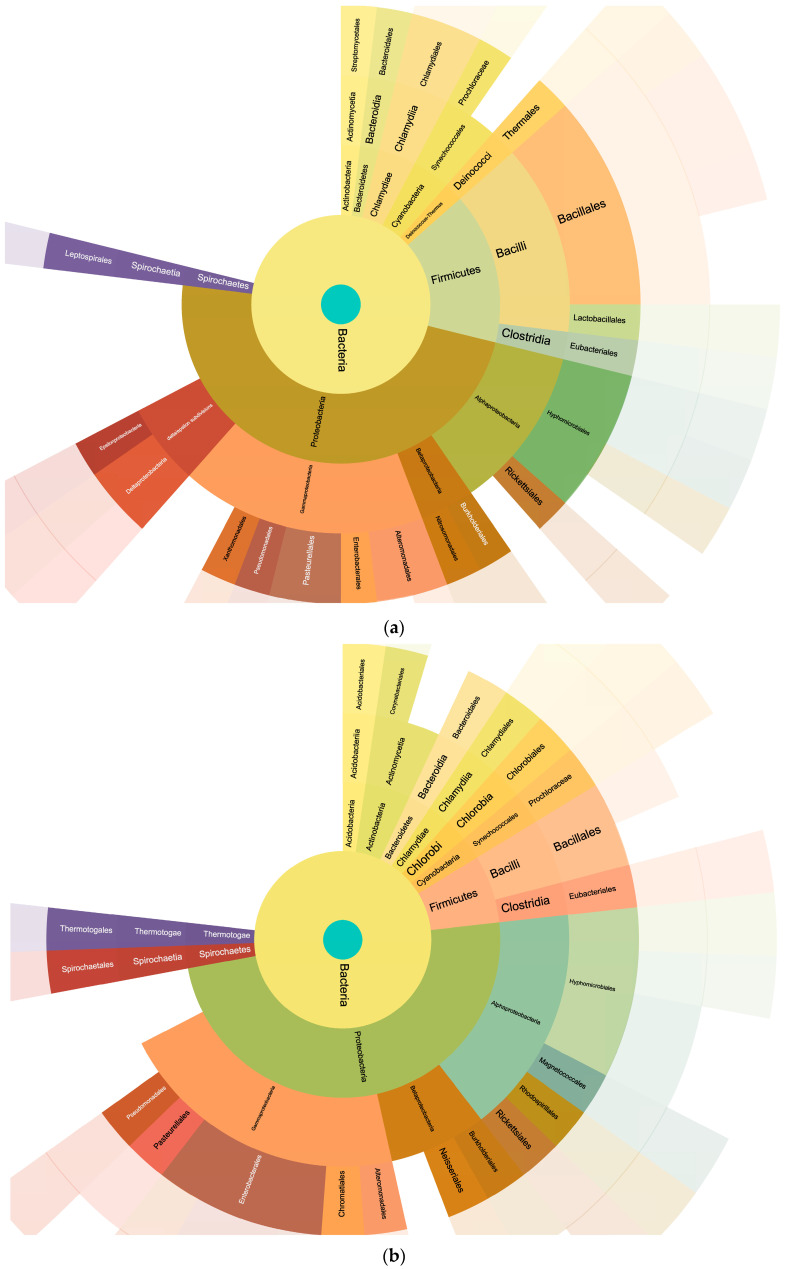

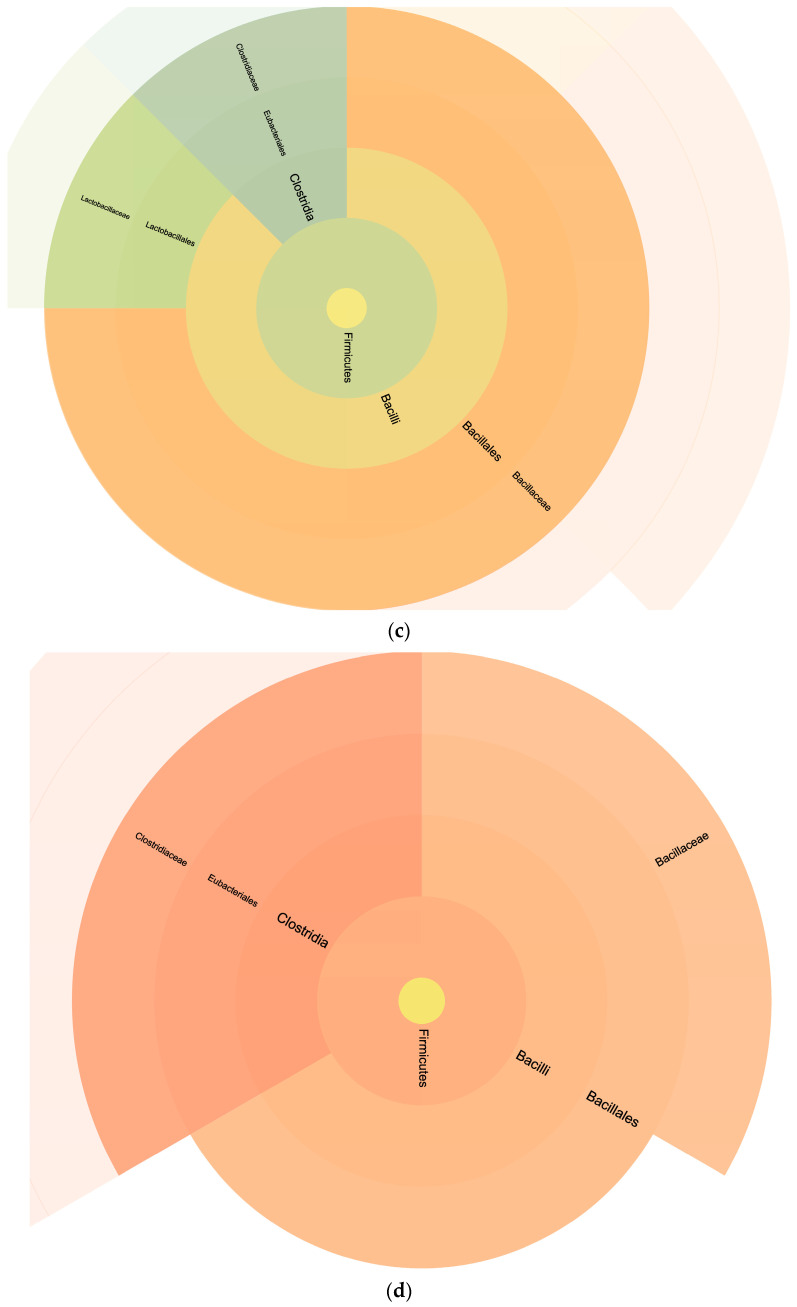
Pie charts detailing the taxonomical classification of the peptides obtained with the MaxQuant searches against the bacteria database. The obtained lists were analyzed with UNIPEPT (see the Section 4) for the creation of the pie charts. Panels (**a**,**c**) represent the control samples. Panels (**b**,**d**) represent the Podolica samples. The vectorial file of this image is uploaded as Supplementary Material (Supplementary Figure S1) and as separated files.

The analysis of the searches performed by using the bacteria database yielded the pie charts shown in Figure 4 detailing the relative percentages of the taxonomy of the identified bacteria.

The bottom-up proteomics datasets were further analyzed using the CARD carrying the known sequences linked to bacterial proteins involved in antimicrobial resistance (see the Data Analysis Section in the methods). As can be seen in Table 2, it was possible to detect beta-lactamases and proteins involved in tetracycline resistance in both the control and Podolica milk samples. Among the detected proteins, only an isoform of beta-lacatamase and an isoform of mosaic tetracycline resistance proteins appeared to be differentially abundant between the control and Podolica groups with the beta-lactamase being more abundant in the Podolica milk samples.

## 3. Discussion

The results obtained from the LAP-MALDI MS coupled with LDA on the training and test sets provide valuable insights into the feasibility and accuracy of differentiating between the Podolica and control milk samples based on their MS profiles [20]. The classification accuracy achieved on the training set, with a correct classification percentage of around 86%, demonstrates the potential of LAP-MALDI MS and LDA in distinguishing between the Podolica and control milk samples and suggests that there are discernible differences in the MS profiles of these two types of milk. However, it is important to acknowledge that there were still instances of misclassification within the training set, with four control samples being erroneously classified as Podolica samples and five Podolica samples misclassified as control samples. The analysis of the test set yielded a correct classification percentage of more than 98% with just one misclassified data point.

This high classification accuracy obtained by the LAP-MALDI MS profiling coupled with LDA suggests that this method is effective in distinguishing between Podolica and control milk samples, as demonstrated by the independent dataset not used during model training. However, it is worth noting that 1 Podolica sample out of 34 was incorrectly classified as a control sample (Table 1). While the overall classification accuracy is commendable, it should be noted that the current dataset is limited with regard to the number of biological replicates. The overall accuracy of the models obtained by this approach can be better established once higher numbers of biological replicates are available.

Figure 2 presents two of the most influential ions for the classification, shedding light on the specific *m*/*z* peaks that contribute significantly to the differentiation between the Podolica and control samples. These peaks correspond to distinct molecular entities that are more abundant in control milk, and less abundant in Podolica milk.

The successful classification of these two types of milk and the detection of a different molecular profile suggested the presence of biochemical differences between the two milk types that were worth further consideration.

Bottom-up proteomics combined with the enrichment of bacterial cells and somatic cells has provided valuable insights into the composition of the milk samples, shedding light on differential representation and the presence of specific proteins. Among the 220 proteins identified using a *Bos taurus* database, cathelicidins and annexins were particularly noteworthy, as shown in Figure 3. These proteins exhibited higher levels of abundance in the milk of control cows.

Cathelicidins are a group of antimicrobial peptides with a well-established role in innate immunity. Their higher abundance in control cows suggests that these animals utilize a greater antimicrobial defense system in their mammary glands. This finding could be related to differences in breed genetics, environmental factors, or other variables that influence the expression of cathelicidins [21,22].

Annexins are a family of calcium-dependent phospholipid-binding proteins involved in various cellular processes, including inflammation and tissue repair. The higher abundance of annexins in control cows suggests potential differences in immune response and tissue maintenance between the two groups [23]. Their pro-inflammatory activity is well documented [24] and is in agreement with an increased overall response against possible pathogens. Overall, these two findings seem to indicate an increased level of inflammation and defense against pathogens in control cows.

The analysis conducted using the FASTA database with bacterial sequences provided an overview of the relative percentages of bacterial taxa detected in the milk samples (Figure 4). This information plays a critical role in unraveling the complex microbial consortia within the milk. Interestingly, the Podolica milk had a lower amount of firmicutes and did not reveal the presence of lactobacilli that were, on the other hand, present in the control milk. The presence of lactobacilli in the control samples and the higher relative abundance of peptides from clostridia in the Podolica milk samples may be related to the different surrounding environments during the milking process [25]. In rural environments, Podolica cows are often milked after the calf begins suckling, with the calf in close proximity to the cow during the milking process. This proximity may result in a higher presence of environmental bacteria. Clostridial presence is usually associated with the phenomenon of late blowing in hard pasta cheese [26,27]. However, the presence of these bacterial species might provide probiotic and anti-inflammatory properties [28,29]. For example, clostridia from clusters XIVa and IV are prevalent in the mouse intestine and play a crucial role in normalizing germ-free (GF) mice [30]. Moreover, clusters XIVa and IV are crucial as competitors for the growth of other pathogens such as *Enterococcus* [31].

Proteobacteria quantity was around 50% in both milk samples, but the control samples showed a lower amount of enterobacteriales and higher amounts of deltaproteobacteria. These results are consistent with the previously published data reporting the percentages of Proteobacteria being from 32.94% to 70.55% and of Firmicutes being from 11.07% to 39.19% [32,33].

In parallel, the Comprehensive Antibiotic Resistance Database (CARD) was employed to identify the known sequences linked to the bacterial proteins involved in AMR. The subsequent analysis, as presented in Table 1, provides insights into the detection and distribution of AMR-specific proteins in both the control and Podolica milk samples.

So far, only beta-lactamases and proteins associated with tetracycline resistance have been detected. Beta-lactamases are enzymes known for their ability to hydrolyze beta-lactam antibiotics, rendering them ineffective [2]. Their abundance was higher in Podolica milk (Supplementary Figure S1). On the other hand, tetracycline resistance proteins confer resistance to tetracycline antibiotics. Their abundance was similar amongst the two experimental groups. The presence of these AMR proteins in the milk samples raises important questions regarding the potential sources and implications of AMR in dairy production; however, in this case, this might be related to the higher heterogeneity of the bacterial population of the milk from Podolica cows [34]. From this perspective, it is known that the environment is a great source of different bacterial species that might carry resistance genes and proteins [35].

It is notable that, with the exception of beta-lactamases, no relevant difference was observed in the occurrence of CARD proteins between the two groups of milk samples. It is possible that the AMR proteins identified in the milk samples originate from bacteria within the mammary gland that are common to both the control and Podolica cows.

Moreover, the absence of a significant difference in AMR protein detection could be attributed to shared environmental and management practices across both groups of cows. Factors such as farm hygiene, antibiotics use, and exposure to resistant bacteria in the surrounding environment can influence the prevalence of AMR. If these factors are consistent between the two groups, it may explain the observed similarities in the AMR protein profiles.

It is important to emphasize that the detection of AMR proteins does not necessarily imply the presence of antibiotic-resistant bacteria in milk. The proteins themselves may be present because of being induced by natural bacterial competition mechanisms [36]. Nevertheless, the identification of AMR proteins serves as an indicator of potential AMR-related concerns in dairy production.

## 4. Materials and Methods

### 4.1. Samples

Eight farms in Calabria (Italy) were included in the study—four Podolica farms and four (control) farms with other cattle breeds.

The study was made on bulk milk samples from the four Podolica farms and four farms with non-Podolica (control) cows (Frisona, Pezzata Rossa, and Bruna Alpina breeds; one farm had mixed Frisona and Pezzata Rossa). From each were collected two samples (bulk tank and herd average) at two different times, at least a week apart, to ensure variability. When the milk collection was scheduled, if a cow received a treatment with antibiotics in the previous two months, it was excluded from that milking session to minimize the eventual effect of recent antibiotic treatments. Podolica herds had 50, 35, 40, and 30 cows, respectively (totaling 155). These cows grazed on Mediterranea pastures without supplementary feed, except for polyphyte hay in the coldest winters (about 3 kg/head/day). During summer, they were moved to mountain pastures at 1500 m above sea level. Control farms were composed as follows: 54 Pezzata Rossa (fed a mixed ration of dehydrated corn), 60 Bruna Alpina (fed ad libitum with sorghum silage), 35 Frisona, and a farm had 40 mixed Frisona and Pezzata Rossa cows (fed corn silage ad libitum with 4.5 kg of hay/day and concentrates).

A total of 250 mL of milk were taken from the top of the tank using a clean, sanitized dipper after the milk was agitated for 5–10 min as described in reference [37]. For each sample, one aliquot of 100 mL was used for somatic cell count (SCC) (Fossomatic; FOSS, Hilleroed, Denmark). The samples were transported at 4 °C to the laboratories of Centro Interdipartimentale di Servizi Veterinari, Magna Graecia University of Catanzaro, Italy, and frozen in different aliquots at −80 °C until further analysis.

### 4.2. Solvents and Reagents

Water and acetonitrile (ACN) (OPTIMA^®^ LC/MS grade) for LC/MS analyses were obtained from Fisher Scientific (Milan, Italy). Isopropanol, α-cyano-4-hydroxycinnamic acid (CHCA), 1,4-dithiothreitol (DTT), Formic Acid (FA), iodoacetamide (IAA), ammonium bicarbonate, and trichloroacetic acid (TCA) were purchased from Sigma-Aldrich, Merck Life Science S.r.l., Milano, Italy. Sequencing-grade modified porcine trypsin (lyophilized) was obtained from Promega, Milano, Italy.

### 4.3. LAP-MALDI MS

LAP-MALDI MS profiling was employed to distinguish 100% Podolica milk from the milk obtained from the other Calabrian cattle breeds. For this analysis, defrosted milk samples underwent a centrifugation step, and the resulting supernatants were processed using TCA precipitation, and subsequently analyzed as previously described [7].

Briefly, the milk samples were thawed at room temperature and kept in an ice bath during aliquoting. Aliquots of 200 µL were subjected to a precipitation step by adding 100 µL of 5% (*w*/*v*) TCA. The samples were then centrifuged at 3000× *g* for 5 min. After discarding the supernatants, the pellets were re-suspended in 1 mL of a water/acetonitrile/isopropanol mixture (1:1:1, *v*/*v*/*v*). Following a 60-s sonication, the samples were stored at −20 °C until the LAP-MALDI sample preparation and MS analysis.

The matrix chromophore CHCA was dissolved in acetonitrile/water (70/30, *v*/*v*) to a final concentration of 30 mg/mL using 2 min of sonication. This solution was then mixed with ethylene glycol in a 10:7 ratio to create the liquid support matrix (LSM) for LAP-MALDI.

The milk sample extracts were thawed at room temperature for 5 min and mixed in a 1:1 ratio with the LSM. Droplets of the resulting sample mixtures (1.2 μL each) were spotted onto a 96-well Waters MALDI sample plate (Waters Corporation, Wilmslow, UK) and analyzed using LAP-MALDI MS on a Synapt G2-Si hybrid quadrupole-time of flight mass spectrometer (Waters Corporation) equipped with ion mobility separation.

The LAP-MALDI source was a custom modification of a Waters electrospray ionization source (Waters Corporation) [7].

### 4.4. Bottom-up Proteomics

#### 4.4.1. Sample Treatment

The protein concentration of each extract was measured using the Qubit Protein Assay kit and the Qubit 1.0 Fluorometer (ThermoFisher Scientific, Milan, Italy). A portion of each extract, containing approximately 50 µg of protein, was treated with 39 µg of DTT for reduction (3 h at 20 °C), followed by alkylation with 94 µg of IAA (1 h in the dark at 20 °C). The samples were then digested with porcine trypsin at an enzyme/substrate ratio of 1:50 (overnight at 37 °C). To achieve a final concentration of 25 ng/µL for each sample, a 5% aqueous formic acid solution was added, bringing the final volume to 2 mL [38].

#### 4.4.2. LC-MS/MS

The MS/MS data were collected using a ThermoFisher Scientific Orbitrap Fusion Tribrid^®^ (Q-OT-qIT) mass spectrometer (ThermoFisher Scientific, Bremen, Germany). Liquid chromatography was performed with a ThermoFisher Scientific Dionex UltiMate 3000 RSLCnano system (Sunnyvale, CA, USA). One microliter of the peptide mixture was loaded onto an Acclaim^®^ Nano Trap C18 Column (100 μm i.d. × 2 cm, 5 μm particle size, 100 Å pore size). After washing the trapping column with solvent A (0.1% FA in H_2_O) for 3 min at a flow rate of 7 μL/min, the peptides were eluted onto a PepMap^®^ RSLC C18 EASYSpray column (75 μm i.d. × 50 cm, 2 μm particle size, 100 Å pore size) and separated at 40 °C with a flow rate of 0.25 μL/min using a linear gradient of solvent B (0.1% FA in ACN) in solvent A. The gradient started with 5% for 3 min, increased from 5% to 20% over 32 min, 20% to 40% over 30 min, 40% to 60% over 20 min, and 60% to 98% over 15 min, followed by holding at 98% for 5 min, then decreasing to 5% over 1 min, and re-equilibrating at 5% for 20 min.

The eluted peptides were ionized via electrospray ionization using a source voltage of 1.75 kV and introduced into the mass spectrometer through a heated ion transfer tube at 275 °C. The survey scans of the peptide precursor ions were performed from *m*/*z* 200 to 1600 at a resolution of 120,000 at *m*/*z* 200. MS/MS was conducted by isolating the precursor ions at 1.6 Th using the quadrupole, followed by HCD fragmentation with a normalized collision energy of 35 eV, and rapid-scan MS fragment ion analysis in the linear ion trap (low-resolution MS/MS). The precursor ions with a charge state of 2-4 and an intensity above 5 × 10^3^ counts were selected for MS/MS. Dynamic exclusion was set to 60 s with a 10 ppm tolerance around the selected precursor ion and its isotopes. Monoisotopic precursor ion selection was enabled. The instrument operated in top-speed mode with 3-s cycles, performing MS/MS continuously until the list of non-excluded precursor ions was exhausted or for a maximum of 3 s. The MS/MS spectral quality was enhanced using the parallelizable time option, maximizing the time for MS/MS precursor ion injection and detection during full-scan detection. The mass spectrometer was calibrated using the Pierce^®^ LTQ Velos ESI Positive Ion Calibration Solution (ThermoFisher Scientific). To assess reproducibility, triplicate RP-nHPLC/nESI-MS/MS analyses were performed for each sample.

### 4.5. Data Analysis

For LAP-MALDI MS, raw MS datasets were processed using Abstract Model Builder (AMX; version 1.0.1563.0; Waters, Milford, MA, USA) for classification analysis. The AMX-processed LAP-MALDI mass spectra were generated by accumulating MS scans within each sample’s retention time window, excluding ion mobility drift time data. Ion intensity data matrices were created by binning mass spectral intensities at 1-Th intervals, normalizing them to the total sum intensity of all the bins, and saving them as .csv files. The AMX software provided “loading plots” that identified the most influential features driving the classification.

These datasets were further analyzed using the JMP software (version 14; SAS Institute Inc., Marlow, UK) with the discriminant analysis (Wide Linear) function. Each machine learning model was trained using the LAP-MALDI MS profiles from one dataset and tested on a separate dataset, both obtained through the same mass spectral acquisition process and following identical sample preparation protocols (see ‘LAP-MALDI MS’ section). After acquiring all the LAP-MALDI MS profiles, a blinded classification analysis was performed, using either fully randomized or representative sampling methods, with randomly selected sample sets per group for training and testing.

LC-MS(/MS) data were initially processed for qualitative analysis using the PEAKS Xpro sequencing software (Bioinformatics Solutions Inc., Waterloo, ON, Canada). The processed data were searched with the integrated search routine of PEAKS Xpro (Version 10) against a Bos taurus protein FASTA database (6936 protein sequences) downloaded from the UniProt database (1 February 2023). The Common Repository of Adventitious Proteins (The Global Proteome Machine, c-RAP, version 1.0, 01/01/2012) contaminant database was also included in the search. Database searching was performed using the following parameters: (i) full tryptic peptides with a maximum of 3 missed cleavage sites; (ii) cysteine carbamidomethylation as a fixed modification; (iii) the oxidation of methionine, and the transformation of N-terminal glutamine and N-terminal glutamic acid residues to pyroglutamic acid as variable modifications. The precursor mass tolerance was set to 10 ppm, and the maximum fragment mass error was set to 0.6 Da. Peptide Spectral Matches (PSMs) were validated using a Target Decoy PSM Validator node based on q-values with a 1% False Discovery Rate (FDR).

The label-free quantification (LFQ) analysis of the MS/MS data was performed using the MaxQuant software (version2.4.2.0). The peak lists were searched against the UniProt *Bos taurus* (same as previously mentioned), bacteria (filtered by taxonomy, reviewed, 336,171 hits, 3 July 2023), or CARD (https://card.mcmaster.ca/browse, 5010 reference sequences, January 2023 [39]) FASTA protein databases. For the searches, the precursor mass tolerance was set to 20 ppm for the initial search and 4.5 ppm for the main search, with a fragment mass tolerance of 0.5 Da. Trypsin/P was specified as the enzyme, allowing for up to two missed cleavages. Variable modifications included the oxidation of methionine (M) and the acetylation of the protein N-terminus. The carbamidomethylation of cysteines was set as a fixed modification. A protein-level FDR of 1% was applied to filter the results. The LFQ minimum number of neighbors was set to 3, with an average of 6 neighbors.

The list of peptides obtained by searching the bacteria database was analyzed with the web metaproteomics tool of UNIPEPT (https://unipept.ugent.be/mpa, accessed date: 29 July 2024) for taxonomical classification.

## 5. Conclusions

In conclusion, this study presents an in-depth investigation into the composition of milk, particularly focusing on antimicrobial peptides (AMPs) and resistome proteins, and their implications for human health and food safety. Advanced analytical techniques, such as LAP-MALDI MS profiling, bottom-up proteomics, and metaproteomics, were employed to analyze milk samples from various bovine breeds, with a primary focus on Podolica milk and its differences from milk from other breeds.

The LAP-MALDI MS profiling results, supported by linear discriminant analysis (LDA), demonstrate the potential to differentiate between Podolica and control milk samples based on MS profiles. The classification accuracy achieved is extremely promising but requires further validation with more biological replicates as well as the consideration of confounding factors.

The ability to accurately classify milk samples from different bovine breeds, such as Podolica vs control, can have significant implications for food quality control, product authenticity, and traceability. It can ensure the integrity of products derived from specific breeds, such as the cheese made exclusively from Podolica milk, and enhance consumer confidence in the authenticity of such products.

Bottom-up proteomics revealed approximately 220 differentially abundant proteins, with cathelicidins and annexins showing higher levels of abundance in control cows.

In more rural settings, diverse plant grazing with a potentially greater variety of plants and the ingestion of antimicrobial plants, lower stress levels, and lower exposure to factors exogenous to the natural environment as well as genetic factors and farm management practices (number of animals per area) may lead to overall better health and a more robust immune system, thus reducing the need for the expression of defense peptides and proteins in milk.

The taxonomic distribution of identified bacteria generally highlights the diversity within the mammary gland’s microbial ecosystem and its link with the surrounding environment.

Analysis using the Comprehensive Antibiotic Resistance Database (CARD) detected the presence of beta-lactamases and tetracycline resistance proteins in both the control and Podolica milk samples, with no significant breed-based differences observed. This underscores the general potential of the routine monitoring of antimicrobial resistance (AMR) in dairy production.

Finally, it can be concluded that Podolica cow milk, being produced in a more ‘rural’ environment, shows a higher presence of clostridia and a lower amount of proteins/peptides with antimicrobial activity compared to cow milk of other breeds.

## Figures and Tables

**Table 1:** Confusion matrices showing the results for both the training set classification as shown in Figure 1 and the test set application using the classification model obtained by the training set.

Training Set			Test Set		
Actual	Predicted Count		Actual	Predicted Count	
Class	Control	Podolica	Class	Control	Podolica
**Control**	28	4	**Control**	32	0
**Podolica**	5	27	**Podolica**	1	31

**Table 2:** LC-MS/MS database search hits obtained from the CARD for the detection of proteins involved in antimicrobial resistance mechanisms.

MAJORITY PROTEIN IDS	PEPTIDE SEQUENCES
GB|AAR29969.1|ARO:3007121|TET(O/W)	VQEASLFPVYHGSAKK
GB|ACH59005.1|ARO:3002513|LRA-19	STNAQLMIDEK
GB|WP_052769157.1|ARO:3005493|IND-16	TMNKLKTK
GB|WP_070210118.1|ARO:3005873|OXA-658	QDASLSSAIKR
GB|AHE40505.1|ARO:3004542|MPHN	IRMEKVR
GB|AKQ05896.1|ARO:3004587|TET(52)	EGQDVEIIR;QTDAVVEVQFKDGR
GB|CAA54269.1|ARO:3007105|NIMC	MFRAMRPK
GB|CAA90891.1|ARO:3004762|BRO-2	MMQRRHFLQK
GB|CAL84423.1|ARO:3004146|CFRC	IDNFHDMHILPK

## Data Availability

The data are contained within the article.

## Supplementary Materials

The following supporting information can be downloaded at: https://www.mdpi.com/article/10.3390/antibiotics13090838/s1, Figure S1: The vectorial file of this Figure 4.
